# Laparoscopic versus open pediatric inguinal hernia repair: state-of-the-art comparison and future perspectives from a meta-analysis

**DOI:** 10.1007/s00464-019-06960-2

**Published:** 2019-07-17

**Authors:** Kelly Dreuning, Sanne Maat, Jos Twisk, Ernest van Heurn, Joep Derikx

**Affiliations:** 1 Department of Pediatric Surgery, Emma Children’s Hospital, Amsterdam UMC, University of Amsterdam & Vrije Universiteit Amsterdam, P.O. Box 22660, 1100 DD Amsterdam, The Netherlands; 2 Department of Methodology and Applied Biostatistics, and the Amsterdam Public Health Research Institute, Amsterdam UMC, Vrije Universiteit, Amsterdam, The Netherlands

**Keywords:** Hernia, inguinal, Hernia repair, Laparoscopy, Child

## Abstract

**Background:**

Laparoscopic inguinal hernia repair in children is increasingly performed as it allows contralateral inspection and potentially results in shorter operation time and less complications. Evidence from meta-analyses of randomized controlled trials (RCTs) regarding the superiority of laparoscopic versus open hernia repair is lacking.

**Methods:**

A systematic literature search was performed querying PubMed, Embase, MEDLINE, and the Cochrane Library databases. RCTs comparing laparoscopic with open hernia repair in children were considered eligible, without year and language restrictions. Cochrane Risk of Bias tool was used for quality assessment. Data were pooled using a random-effects model. Subgroup analyses were performed according to the laparoscopic suturing technique (i.e., intracorporeal or extracorporeal).

**Results:**

Eight RCTs (*n* = 733 patients; age range 4 months–16 years) were included in this meta-analysis. Laparoscopic (LH) and open (OH) hernia repair was performed in 375 and 358 patients, respectively. Complications (seven RCTs, *n *= 693; pooled OR 0.50, 95% CI 0.14 to 1.79), recurrences (seven RCTs, *n *= 693; pooled OR 0.88, 95% CI 0.20 to 3.88), and MCIH rates (four RCTs, *n *= 343; pooled OR 0.28, 95% CI 0.04 to 1.86) were not different between the groups. LH resulted in shorter bilateral operation time (Five RCTs, *n *= 194; weighted mean difference (WMD) − 7.19, 95% CI − 10.04 to − 4.34). Unilateral operation time, length of hospital stay, and time to recovery were similar. There was insufficient evidence to assess postoperative pain and wound cosmesis, and evidence of substantial heterogeneity between the included studies. Subgroup analyses demonstrated less complications and shorter unilateral operation time for extracorporeal suturing and shorter length of hospital stay for intracorporeal suturing.

**Conclusions and relevance:**

No definite conclusions to decide on the superiority of one of either treatment strategies can yet be drawn from the available literature. There was evidence of substantial heterogeneity and the clinical relevance of most estimated effects is very limited.

**Electronic supplementary material:**

The online version of this article (10.1007/s00464-019-06960-2) contains supplementary material, which is available to authorized users.

The incidence of pediatric inguinal hernia ranges from 0.8 to 5% and increases to more than 30% in preterm born infants [1, 2]. Treatment is necessary because of the risk of incarceration of bowel, testis, or ovary, which occurs in approximately 3–16% of children with inguinal hernia [2, 3]. Open inguinal hernia repair is the most performed treatment strategy in children; however, the laparoscopic approach is increasingly used in current practice [4]. Although inguinal hernia repair is the most commonly performed operation by pediatric surgeons, there still is no clear consensus which technique is superior in children who need to undergo inguinal hernia repair: the open or laparoscopic.

Laparoscopic hernia repair allows better visualization of the inguinal region thereby enabling detection of a contralateral patent processus vaginalis (CPPV), which can be simultaneously closed since the presence of a CPPV might result in development of a metachronous contralateral inguinal hernia (MCIH). Open repair offers the possibility for loco regional (caudal) anesthesia, which might be beneficial as repeated or prolonged general anesthesia carries risks for near critical incidents and the U.S. Food and Drug Administration (FDA) recently released a warning that repeated or prolonged general anesthesia potentially harms the child’s developmental brain [5, 6]. In 2016, the International Pediatric Endosurgery Group (IPEG) reviewed all existing evidence on minimal access approaches in the treatment of pediatric inguinal hernia and concluded that laparoscopic hernia repair resulted in shorter operation time for bilateral hernia repair and less postoperative complications compared to the open technique [7]. Conversely, there was also a trend towards higher recurrence rates in laparoscopic hernia repair [8].

Several systematic reviews comparing laparoscopic with open pediatric hernia repair have previously been published [8–11], although the number of studies providing level 1a evidence is very limited. Moreover, many outcome parameters have not been addressed [11]. Consequently, there still is an ongoing debate about the best treatment strategy and decisive evidence on the superiority of one of either treatment strategies is lacking. The aim of this systematic review and meta-analysis is to provide an extensive state-of-the-art comparison and overview on high-level evidence for most relevant outcome measures including operative and postoperative complications, duration of surgery and hospital admission, postoperative pain, time to full recovery, recurrence rate, MCIH rate, cosmetic appearance, and health care costs.

## Materials and methods

### Literature search

A systematic review and meta-analysis was conducted according to the Preferred Reporting Items for Systematic Reviews and Meta-Analyses (PRISMA) statement. The protocol was registered in PROSPERO (CRD42018116953). An extensive literature search was performed in November 2017 and updated in August 2018 using PubMed, EMBASE, MEDLINE, and the Cochrane Library databases (see Search strategy, Supplementary Material 1). All studies that compared open versus laparoscopic hernia repair in children with inguinal hernia were considered eligible for inclusion, and no year or language restrictions were applied. Reference lists of eligible articles were also queried. The following subject headings (MeSH) and text words were used: inguinal hernia, children/child, p(a)ediatric, laparoscopic/laparoscopy. Institutional Review Board (IRB) approval and written consent were not required for conducting this meta-analysis.

### Eligibility criteria

In this review, all available randomized controlled trials (RCTs) that compared open with laparoscopic hernia repair in children (younger than 18 years old) with inguinal hernia were considered eligible. Only RCTs were included to achieve the highest level of evidence; all other study designs were excluded. Primary outcome measures included operative (i.e., injury of spermatic vessels or spermatic cord, tuba lesions, bleeding, and apnea) and postoperative complications (i.e., hematoma/scrotal edema, hydrocele, wound infection, iatrogenic ascent of the testis, and testicular atrophy). Secondary outcome measures were duration of surgery, length of hospital stay, postoperative pain (pain scores and pain-medication requirement), return to full recovery, recurrence, MCIH, and cosmetic results.

### Study selection and methodological quality assessment

The screening and selection of studies based on title and abstract (level 1), full-text screening (level 2), and quality assessment were independently performed by two review authors (SM and KD). Risk of bias was assessed by the two review authors using the Cochrane Risk of Bias tool for Randomized Controlled Trials. Inconsistencies were solved by second joint review of the literature or by consulting a third independent review author (JD).

### Data extraction

Supplementary Material 2 comprises systematically extracted data regarding important study details and patient characteristics from the included studies. Missing data were retrieved by contacting the study author(s) and/or calculated if possible. In case of any disagreement by the two reviewers, a third reviewer (JD) was consulted after joint review, literature review, and discussion.

Different techniques are currently used for laparoscopic repair of pediatric inguinal hernia. Therefore, we categorized the laparoscopic techniques according to the method that was used to close the internal ring: intracorporeal suturing (intracorporeal) or by placing the suture through the abdominal wall (extracorporeal).

### Statistical analysis

Statistical analysis was performed using Review Manager (Version 5.3. Copenhagen: The Nordic Cochrane Centre, The Cochrane Collaboration, 2014). Heterogeneity was assessed using the *I*^2^ statistic. Meta-analyses were performed using a random-effects model. Weighted (WMD) or standardized (SMD) mean differences and odds ratios (OR) with their corresponding 95% confidence intervals (CI’s) were used for the analysis of continuous and dichotomous variables, respectively. Subgroup analyses were performed to address whether the summary effects vary between different laparoscopic techniques, as differences in laparoscopic suturing technique (i.e., intracorporeal suturing and extracorporeal suturing) may modify the effect of the intervention. Sensitivity analyses were conducted to examine the treatment effects caused by studies with high risk of bias regarding the selection of patients (i.e., inclusion of exclusively boys). Regarding the development of MCIH, sensitivity analysis was performed by excluding studies that did not simultaneously close a laparoscopically detected CPPV during unilateral hernia repair.

## Results

### Literature search

The search strategy yielded 674 potentially eligible articles after removal of duplicates. After the initial screening by title and abstract, 32 full-text articles were assessed for eligibility (see PRISMA flow chart, Supplementary Material 3). Twenty-four studies were excluded as they did not meet the inclusion criteria. After translation of one Turkish and one Chinese article, eight randomized controlled trials (*n *= 733) were included in this review and meta-analysis [12–19].

### Study characteristics

The eight randomized controlled trials were published between 2005 and 2016 (Table 1). The total study population consisted of 733 children with inguinal hernia: 375 children underwent laparoscopic hernia repair (LH) and 358 children underwent open hernia repair (OH). Laparoscopic hernia repair with intracorporeal suturing was performed in 171 patients, laparoscopic repair with extracorporeal suturing in 204 patients. All children received general anesthesia. Unilateral hernia repair was performed in 434 children, bilateral hernia repair in 194 children, and laterality of the inguinal hernia was not further specified in 27 patients with recurrent inguinal hernia, 40 patients with inguinal hernia and umbilical hernia, and 38 patients with inguinal hernia and questionable other side [18]. Except from two studies [12, 15], the study population consisted of both male and female pediatric patients. Age and mean follow-up time ranged from 4 months to 16 years and 24 h to 2 years, respectively.

**Table 1:** Summarized study details of the studies included in this meta-analysis

Author	Year	Country	Study design	Patients no. (LH, OH)	Unilateral no. (%)	Bilateral no. (%)	Male no. (%)	Female no. (%)	Age range	Follow-up Mean (SD)/(range)
Celebi et al.	2014	Turkey	RCT	59 (28, 31)	0 (0)	59 (100)	59 (100)	0 (0)	> 6 yr	3–24 mo
Chan et al.	2005	Hong Kong	RCT	83 (41,42)	80 (96.4)	3 (3.6)	67 (80.7)	16 (19.3)	3 mo–18 yr	LH: 12.2 (2.8) mo OH: 11.8 (2.5) mo
Gause et al.	2016	USA	RCT	41 (26, 15)	27 (65.9)	14 (34.1)	31 (75.6)	10 (24.4)	< 3 yr	2 yr
Inal et al.	2013	Turkey	RCT	40 (20, 20)	40 (100)	0 (0)	40 (100)	0 (0)	7–14 yr	24 h
Koivusalo et al.	2008	Finland	RCT	89 (47, 42)	89 (100)	0 (0)	66 (74.2)	23 (25.8)	4 mo–16 yr	24 mo
Saranga et al.	2008	India	RCT	69 (35, 34)^a^	69 (100)	0 (0)	62 (89.9)	7 (10.1)	< 14 yr	3.5 mo
Shalaby et al.	2012	Egypt	RCT	250 (125, 125)^b^	53 (21.2)	92 (36.8)	179 (71.6)	71 (28.4)	14–96 mo	24 (16–30) mo
Zhu et al.	2015	China	RCT	102 (53, 49)	76 (74.5)	26 (25.5)	71 (69.6)	31 (30.4)	7–63 mo	6 mo

*RCT* randomized controlled trial, *LH* laparoscopic hernia repair, *OH* open hernia repair, *SD* standard deviation, *mo* months, *yr* year, *h* hours

^a^In the laparoscopic group (*n *= 51), six had bilateral hernias and ten contralateral patency’s of the processus vaginalis (CPPV) were detected intra-operatively and repaired simultaneously. These 16 bilateral hernia repairs were excluded from comparative analysis and are not included in this table

^b^In addition to patients who presented with (i) unilateral inguinal hernia in obese children (*n *= 53) and (ii) bilateral inguinal hernia (*n *= 92), Shalaby et al. also included patients with (iii) recurrent inguinal hernia (*n *= 27), (iv) inguinal hernia with umbilical hernia and (*n *= 40), (v) inguinal hernia with questionable other side (*n *= 38)

### Methodological quality

Quality assessment using the Cochrane Risk of Bias tool for Randomized Controlled Trials showed that all trials were at low risk of bias for incomplete outcome data (see risk of bias graph and risk of bias summary, Supplementary Material 4). 75% of the trials were at low risk of bias for random sequence generation and allocation concealment. Saranga et al. introduced risk of bias by enrolling patients based on the day they visited the outpatient clinic [17]. Only 50% of the trials were at low risk of bias for blinding of participants and personnel and blinding of outcome assessment. Other risk of bias, e.g., selection bias as only boys were included [12, 15], was considered high in 25%, and risk of bias concerning selective reporting could not be assessed.

### Primary outcome: operative and postoperative complications

**Fig. 1:**
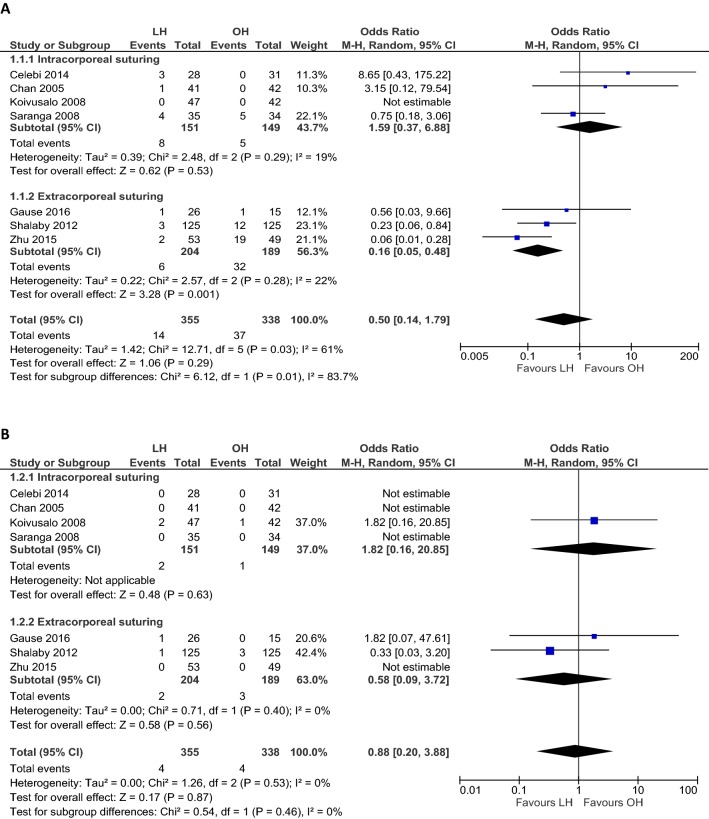

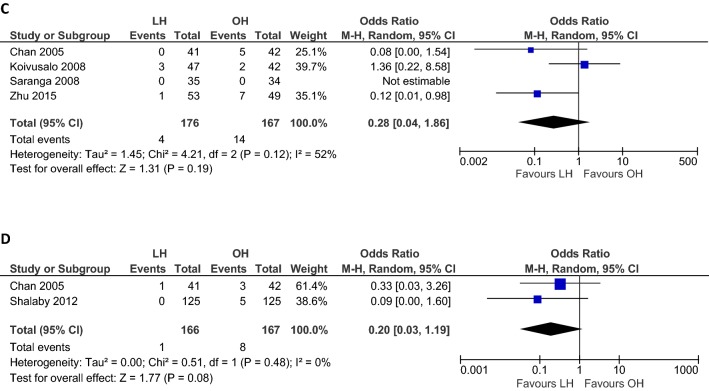
Meta-analysis of operative and postoperative complications, recurrence rate and metachronous contralateral inguinal hernia (MCIH) rate, and cosmetic problems between laparoscopic (LH) and open (OH) inguinal hernia repair. **A** Operative and postoperative complications; **B** recurrence; **C** MCIH; **D** problems with wound cosmesis. Proportionally sized boxes represent the weight of each study; diamond shows the pooled odds ratio; *LH* laparoscopic hernia repair, *OH* open hernia repair, *M–H*, Mantel–Haenszel, *CI* confidence interval

Seven RCTs (*n *= 693) assessed operative and/or postoperative complications and were included in this part of the meta-analysis. There is a large variety in the kind of complications that were analyzed and no study assessed the risk for near critical incidents, i.e., apneas. Operative complications (i.e., injury of spermatic vessels or spermatic cord, tuba lesions, and bleeding) were only reported in the LH group (Table 2). Overall, the complication rate did not differ between laparoscopic and open hernia repair (OR 0.50, 95% CI 0.14 to 1.79; *p *= 0.29; *I*^2^ = 61%; Fig. 1A). A sensitivity analysis including only studies with low risk of bias due to the selection of patients (no restrictions for gender) did not alter the results (*n *= 634; OR 0.33, 95% CI 0.10 to 1.06; *p *= 0.06; *I*^2^ = 51%; Table 3). We found improved heterogeneity, but no difference in complications was observed (OR 1.59, 95% CI 0.37 to 6.88; *p *= 0.53; *I*^2^ = 19%; Fig. 1A) when we included only studies that used intracorporeal suturing as closing method for the laparoscopic technique. If only studies were included that used extracorporeal suturing, heterogeneity was improved and lower complication rates were found for laparoscopic repair (OR 0.16, 95% CI 0.05 to 0.48; *p *= 0.001; *I*^2^ = 22%; Fig. 1A). Two studies reported that three (10.7%) and eight (17%) patients in the LH group postoperatively experienced shoulder pain [12, 16].

**Table 2:** Perioperative and postoperative complications, recurrence, metachronous contralateral inguinal hernia (MCIH) rate, and presence of a contralateral patent processus vaginalis (CPPV)

Author year	Procedure no.	Perioperative complications			Postoperative complications					Recurrence no. (%)	MCIH rate no. (%)	Presence of CPPV no. (%)
		Injury spermatic vessels/vas deferens no. (%)	Ovarian lesion no. (%)	Bleeding no. (%)	Hematoma/edema no. (%)	Hydrocele no. (%)	Wound infection no. (%)	Iatrogenic testicular ascent no. (%)	Testicular atrophy no. (%)			
Celebi 2014	LH: 28	0 (0)	NA	–	0 (0)	3 (10.7)	–	0 (0)	0 (0)	0 (0)	–	–
	OH: 31	0 (0)	NA	–	0 (0)	0 (0)	–	0 (0)	0 (0)	0 (0)	–	NA
Chan 2005	LH: 41	–	–	–	–	1 (2.4)	–	0 (0)	0 (0)	0 (0)	0 (0)	12 (29.3)
	OH: 42	–	–	–	–	0 (0)	–	0 (0)	0 (0)	0 (0)	5 (11.9)	NA
Gause 2016	LH: 26	0 (0)	0 (0)	1 (3.8)^b^	0 (0)	0 (0)	0 (0)	–	0 (0)	1 (3.8)	–	–
	OH: 15	0 (0)	0 (0)	0 (0)	0 (0)	0 (0)	1 (6.7)	–	0 (0)	0 (0)	–	NA
Inal 2013	LH: 20	–	NA	–	–	–	–	–	–	–	–	–
	OH: 20	–	NA	–	–	–	–	–	–	–	–	NA
Koivusalo 2008	LH: 47	–	–	–	–	–	–	0 (0)	0 (0)	2 (4.3)	3 (6.4)	12 (26)
	OH: 42	–	–	–	–	–	–	0 (0)	0 (0)	1 (2.4)	2 (4.8)	NA
Saranga 2008	LH: 35	–	–	2 (5.7)^c^	0 (0)	2 (5.7)	0 (0)	–	–	0 (0)	0 (0)	10 (22.2)
	OH: 34	–	–	0 (0)	2 (5.9)	1 (2.9)	2 (5.9)	–	–	0 (0)	0 (0)	NA
Shalaby^a^ 2012	LH: 125	0 (0)	0 (0)	0 (0)	–	3/87 (3.4)	–	0 (0)	0 (0)	1 (0.8)	–	–
	OH: 125	0 (0)	0 (0)	0 (0)	–	5/92 (5.4)	–	4/92 (4.4)	3/92 (3.3)	3 (2.4)	–	NA
Zhu 2015	LH: 53	–	–	–	2 (3.8)	–	–	–	–	0 (0)	1 (1.9)	Unclear^d^
	OH: 49	–	–	–	19 (38.8)	–	–	–	–	0 (0)	7 (14.3)	NA

*NA* not applicable, *MCIH* metachronous contralateral inguinal hernia, *CPPV* contralateral patent processus vaginalis

^a^Shalaby assessed the occurrence of hydroceles, iatrogenic ascent, and testicular atrophy in the males of both groups

^b^Peritoneal bleeding due to needle injury

^c^Minor bleeding at incision site

^d^Zhu et al. stated that a contralateral processus vaginalis was simultaneously closed when it was found to be patent

**Table 3:** Meta-analysis and sensitivity analyses of laparoscopic versus open inguinal hernia repair in children

Outcome	Studies, *n*	Total participants, *n*	Participants in LH group, *n*	Participants in OH group, *n*	Heterogeneity *I*^2^, %	Mean difference (95% CI)	Odds ratio (95% CI)	*p* Value
Complications	7 [12–14, 16–19]	693	355	338	61		0.50 (0.14 to 1.79)	0.29
Complications (only studies with low risk of bias on patient selection)	6 [13, 14, 16–19]	634	327	307	51		0.33 (0.10 to 1.06)	0.06
Recurrence	7 [12–14, 16–19]	693	355	338	0		0.88 (0.20 to 3.88)	0.87
Recurrence (only studies with low risk of bias on patient selection)	6 [13, 14, 16–19]	634	327	307	0		0.88 (0.20 to 3.88)	0.87
MCIH rate	4 [13, 16, 17, 19]	343	176	167	52		0.28 (0.04 to 1.86)	0.19
MCIH rate (only studies that closed a laparoscopically detected CPPV)	3 [13, 17, 19]	254	129	125	0		0.10 (0.02 to 0.58)	0.01
Unilateral operation time	7 [13–19]	434	226	208	97	0.62 (− 5.70 to 6.95)		0.85
Unilateral operation time (only studies with low risk of bias on patient selection)	6 [13, 14, 16–19]	394	206	188	97	− 0.72 (− 7.53 to 6.09)		0.84
Bilateral operation time	5 [12–14, 18, 19]	194	93	101	73	− 7.19 (− 10.04 to − 4.34)		< .001
Bilateral operation time (only studies with low risk of bias on patient selection)	4 [13, 14, 18, 19]	135	65	70	74	− 7.90 (− 12.49 to − 3.31)		< .001

*LH* laparoscopic hernia repair, *OH* open hernia repair, *CI* confidence interval

### Ipsilateral recurrence rate and MCIH

The recurrence rate (assessed by seven RCTs, *n *= 693) (OR 0.88, 95% CI 0.20 to 3.88; *p *= 0.87; *I*^2^ = 0%; Fig. 1B) and MCIH rate (assessed by four RCTs, *n *= 343) (OR 0.28, 95% CI 0.04 to 1.86; *p *= 0.19; *I*^2^ = 52%; Fig. 1C) were not different between both groups (Table 2). Subgroup analysis for recurrence rate including only studies that performed intracorporeal suturing (OR 1.82, 95% CI 0.16 to 20.85; *p *= 0.63; Fig. 1B) and extracorporeal suturing (OR 0.58, 95% CI 0.09 to 3.72; *p *= 0.56; *I*^2^ = 0%; Fig. 1B) did not change the results. Sensitivity analysis for MCIH rate including only studies that closed a laparoscopically detected CPPV (*n *= 254) resulted in a lower MCIH rate in the LH group compared to the OH group (OR 0.10, 95% CI 0.02 to 0.58; *p *= 0.01; *I*^2^ = 0%, Table 3).

### Operation time

**Fig. 2:**
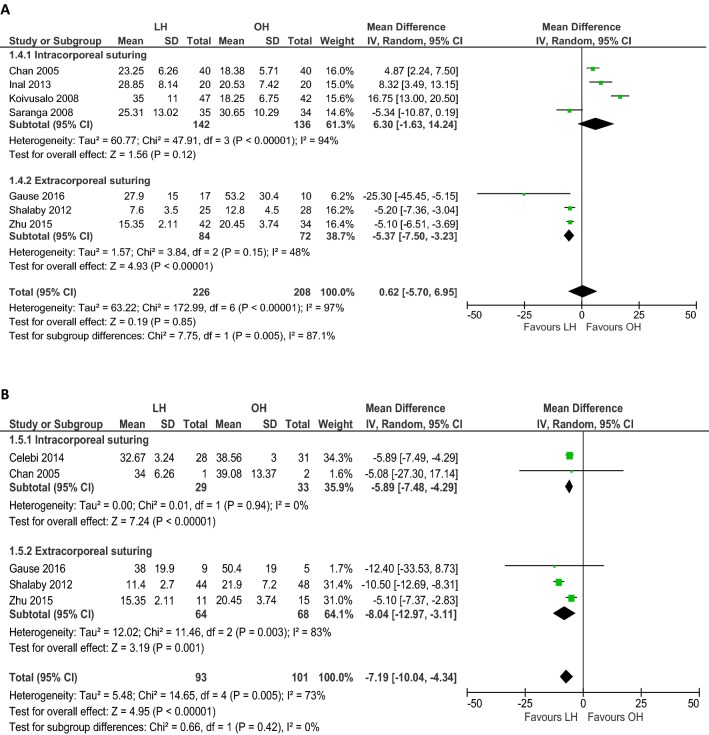

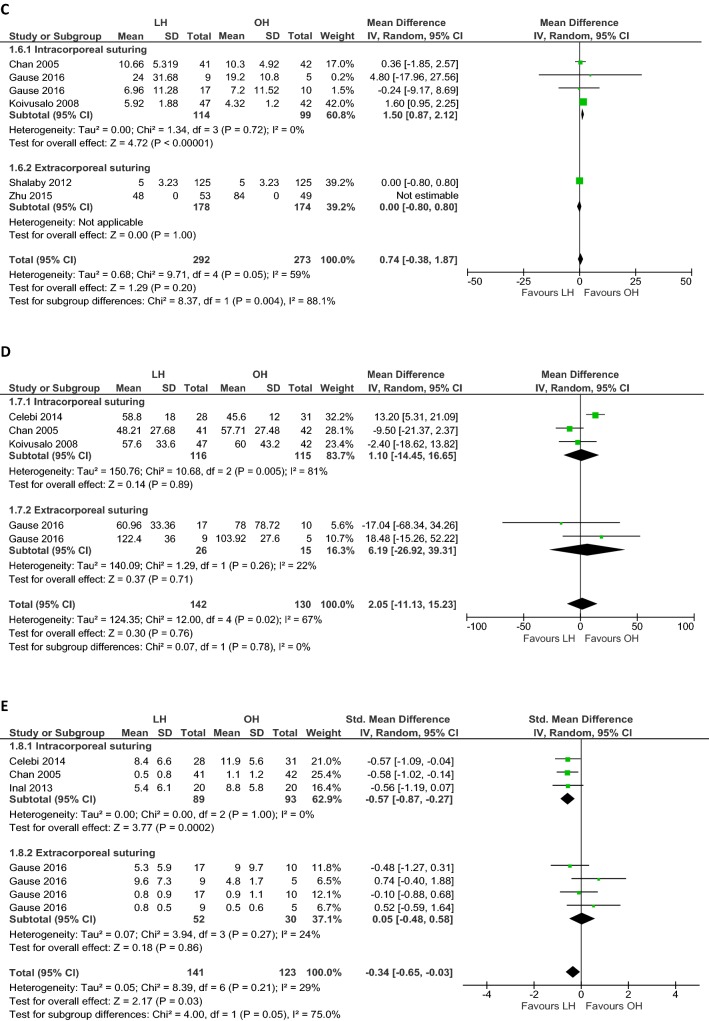

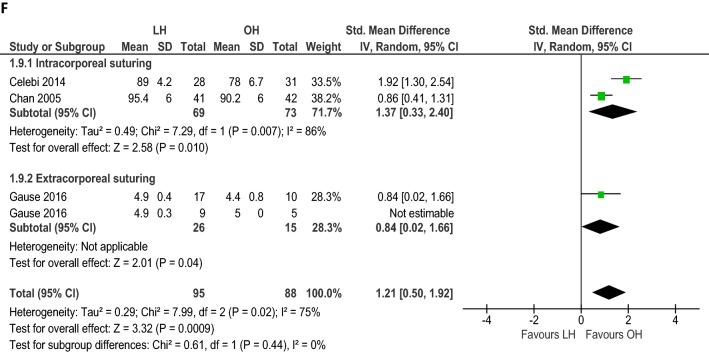
Meta-analysis of continuous outcomes between laparoscopic versus open inguinal hernia repair. **A** Operation time (min) unilateral hernia repair; **B** operation time (min) bilateral hernia repair; **C** length of hospital stay (h); **D** time to full recovery (h); **E** doses of pain medication administered; **F** cosmetic appearance; proportionally sized boxes represent the weight of each study; diamond shows the pooled weighted mean difference; *LH* laparoscopic hernia repair, *OH* open hernia repair, *IV* inverse variance, *CI* confidence interval

Seven RCTs (*n *= 434) reported mean operation times for unilateral hernia repair and five (*n *= 194) for bilateral hernia repair (Table 4). Overall unilateral operation time (min) was not different between LH and OH (WMD 0.62, 95% CI − 5.70 to 6.95; *p *= 0.85; Fig. 2A) with evidence of considerable heterogeneity (*I*^2^= 97%). Similar results were found when we included only studies that performed intracorporeal suturing (WMD 6.30, 95% CI − 1.63 to 14.24; *p *= 0.12; *I*^2^ = 94%; Fig. 2A). Heterogeneity improved and shorter unilateral operation time was found when only studies that performed extracorporeal suturing were included (WMD − 5.37, 95% CI − 7.50 to − 3.23; *p *< 0.001; *I*^2^ = 48%; Fig. 2A). Sensitivity analysis including only studies without gender restrictions (*n *= 394) did not change the outcome (WMD − 0.72, 95% CI − 7.50 to − 3.23; *p *= 0.84; *I*^2^ = 97%; Table 3).

**Table 4:** Intervention characteristics of the included studies

Author year	Closing technique	Trocars	Unilateral operation time, mean (SD), min		Bilateral operation time, mean (SD), min		Length of hospital stay, mean (SD), h		Return to full recovery, mean (SD), h	
			LH	OH	LH	OH	LH	OH	LH	OH
Celebi 2014	Intracorporeal purse-string suture	Two 3 mm One 5 mm	NA	NA	32.7 (3.2)	38.6 (3)	< 24	< 24	58.8 (18)	45.6 (12)
Chan 2005	Intracorporeal purse-string suture	Two 3 mm One 5 mm	23.3 (6.3)	18.4 (5.7)	34 (6.3)	39.1 (13.4)	10.7 (5.3)	10.3 (4.9)	48.2 (28.7)	57.7 (27.5)
Gause 2016	Extracorporeal ligation (SEAL)	Unclear	27.9 (15)	53.2 (30.4)	38 (19.9)	50.4 (19)	Uni: 7 (11.3) Bil: 24 (31.7)	Uni: 7.2 (11.5) Bil: 19.2 (10.8)	Uni: 61 (33.4) Bil: 122.4 (36)	Uni: 78 (78.7) Bil: 103.9 (27.6)
Inal 2013	Intracorporeal purse-string suture	Two 3 mm One 5 mm	28.9 (8.1)	20.5 (7.4)	NA	NA	–	–	7.5 (0.4)	5 (1.4)
Koivusalo 2008	Intracorporeal “N”-shaped suture	Three 5 mm	35 (11)	18.3 (6.8)	NA	NA	5.9 (1.9)	4.3 (1.2)	57.6 (33.6)	60 (43.2)
Saranga 2008	Intracorporeal “Z” suture	Three 5 mm	25.3 (13)	30.7 (10.3)	–	–	< 10 (88.6%) 24 (11.4%)	< 10 (97.1%) 24 (2.9%)	–	–
Shalaby 2012	Extracorporeal suturing with Reverdin needle	One 3 mm	7.6 (3.5)^a^	12.8 (4.5)	11.4 (2.7)	21.9 (7.2)	5 (3.2)	5 (3.2)	<6	<10
Zhu 2015	Extracorporeal suturing with epidural needle	One 3 mm Two 5 mm	15.4 (2.1)^b^	20.5 (3.7)	15.4 (2.1)	20.5 (3.7)	48	84	–	–

*SD* standard deviation, *min* minutes, *mm* millimeter, *LH* laparoscopic hernia repair, *OH* open hernia repair, *uni* unilateral, *bil* bilateral, *SEAL* subcutaneous endoscopically assisted ligation, *NA* not applicable

^a^Only applicable to patients with unilateral and recurrent unilateral hernia; mean (SD) operation time of obese children with inguinal hernia was 9.2 (4.6) min in the LH group and 14.3 (3.6) min in the OH group

^b^Zhu et al. reported the operation time for unilateral and bilateral hernia repair together

Overall duration of laparoscopic bilateral hernia repair (min) was shorter compared to open bilateral hernia repair (WMD − 7.19, 95% CI − 10.04 to − 4.34; *p *< 0.001; Fig. 2B), though again there was evidence of substantial heterogeneity (*I*^2^ = 73%). This effect was still present when we assessed only studies that used the intracorporeal closing technique (WMD − 5.89, 95% CI − 7.48 to − 4.29; *p *< 0.001; *I*^2^ = 0%; Fig. 2B) or the extracorporeal closing technique (WMD − 8.04, 95% CI − 12.97 to − 3.11; *p *< 0.001; *I*^2^ = 83%; Fig. 2B). In sensitivity analysis including only studies without gender restrictions (*n *= 135), these findings proved robust (WMD − 7.90, 95% CI − 12.49 to − 3.31; *p *< 0.001; *I*^2^ = 74%; Table 3).

### Length of hospital stay

Seven RCTs compared the length of hospital stay, i.e., mean time to discharge (h), between LH and OH (Table 4). However, only five studies (*n *= 565) were included in this part of the meta-analysis since mean values could not be retrieved or calculated in two studies which only stated that patients were discharged within a specific time frame after surgery [12, 17]. The length of hospital stay (h) in the LH group was not different from the OH group (WMD 0.74, 95% CI − 0.38 to 1.87; *p *= 0.20; Fig. 2C), with moderate to substantial heterogeneity (*I*^2^ = 59%). Assessing only studies that performed intracorporeal suturing, heterogeneity improved and shorter length of hospital stay was observed in the LH group (WMD 1.50, 95% CI 0.87 to 2.12; *p *< 0.001; *I*^2^ = 0%; Fig. 2C).

### Time to full recovery

Four RCTs assessed the time to full recovery, i.e., time to resume full activity [12–14] or time to normal daily activities [16]. Two studies were excluded from this part of the meta-analysis, as Inal et al. assessed the time to first walking [15] and Shalaby et al. only stated that the time to full recovery was < 6 h (LH group) and < 10 h (OH group) [18]. Analysis of pooled data in four RCTs (*n *= 272) showed that the overall time to return to full recovery (h) was not different between the groups (WMD 2.05, 95% CI − 11.13 to 15.23; *p* = 0.76; *I*^2^= 67%; Fig. 2D) (Table 4). Subgroup analysis on intracorporeal and extracorporeal suturing did not change the outcome (Fig. 2D).

### Postoperative pain and pain-medication requirement

Six studies (*n *= 381 patients) assessed postoperative pain and the need for administering pain medication. Different strategies were used to measure the amount of pain [e.g., Visual Analogue Scale (VAS); children and infants postoperative pain score (CHIPPS); calculation of medication doses administered] and numerous pain medications were prescribed [e.g., patient controlled analgesia (PCA) with bolus morphine; acetaminophen] (Table 5). Since various pain management protocols were used (conceptual heterogeneity), the standardized mean difference was calculated using a random-effects model including four RCTs (*n *= 264). Equal doses of pain medication were administered to patients in both groups (SMD − 0.34, 95% CI − 0.65 to − 0.03; *p* = 0.15; *I*^2^= 29%; Fig. 2E). Including only laparoscopic studies that used intracorporeal suturing, improved heterogeneity and less administration of pain medication were observed (SMD − 0.57, 95% CI − 0.87 to − 0.27; *p* < 0.001; *I*^2^= 0%; Fig. 2E).

**Table 5:** Postoperative pain management, including pain scores and requirement of pain-medication

Author	Pain assessment	Determine severity of pain	Administration	Timing of pain medication	Pain medication	Patients requiring medication		Doses administered/requested^d^	
						LH	OH	LH	OH
Celebi et al.	VAS	VAS (0–10)	Self-administration	During admission	PCA with bolus morphine 10 µg/kg, median (SD)	–	–	A: 8.4 (6.6) R: 8.8 (6.6)	A: 11.9 (5.6) R: 12.5 (10.2)
				After discharge	Ibuprofen 20 mg/kg, median (SD)	–	–	0.8 (0.8)	1.3 (1.2)
Chan et al.	- CHIPPS - CHEOPS	CHIPPS/CHEOPS	CHIPPS ≥ 4 CHEOPS ≥ 5	During admission	Acetaminophen (dose/patient), mean (SD)	–	–	0.5 (0.8)	1.1 (1.2)
Gause et al.	FLACC	–	FLACC > 4	During admission	Acetaminophen 15 mg/kg, mean (SD)	–	–	Uni: 5.3 (5.9) Bil: 9.6 (7.3)	Uni: 9 (9.7) Bil: 4.8 (1.7)
				During admission	Fentanyl 0.5 µg/kg^c^ % patients	Uni: 50% Bil: 75%	Uni: 57.1% Bil: 50%	Uni: 0.8 (0.9) Bil: 0.8 (0.5)	Uni: 0.9 (1.1) Bil: 0.5 (0.6)
Inal et al.	VAS	VAS (0–10)	Self-administration	During admission	PCA with bolus morphine 10 µg/kg, mean (SD)	–	–	A: 5.4 (6.1) R: 10.7 (7.3)	A: 8.8 (5.8) R: 33.2 (6.2)
Koivusalo et al.	- Modified OPS - Pain scale^a^	OPS (0–9) Pain Scale (0–3)	Judged by attending nurse^b^	During admission	Fentanyl 1.0 µg, No. (%) patients	37 (79)	20 (48)	37 (79)	20 (48)
				After discharge	Ibuprofen 20 mg/kg, median (range)	–	–	1 (0–3)	1 (0–5)
Saranga et al.	- CHIPPS - CHEOPS - VRS	- Nil - Mild - Moderate	Unclear	During admission	Acetaminophen 15 mg/kg, No. (%) patients - Nil pain - Mild pain - Moderate pain	2 (6) 30 (86) 3 (8)	0 (0) 32 (94) 2 (6)	–	–
Shalaby et al.	–	–	–	–	–	–	–	–	–
Zhu et al.	–	–	–	–	–	–	–	–	–

*VAS* visual analogue scale, *CHIPPS* children and infants postoperative pain score (< 3 years), *CHEOPS* Children’s Hospital of Eastern Ontario Pain Scale (> 3 years), *FLACC* face, legs, activity, cry, consolability scale, *OPS* objective pain scale, *VRS* verbal response score, *PCA* patient controlled analgesia, *PCM* paracetamol, *iv* intravenous, *Uni* unilateral, *Bil* bilateral

^a^No pain = 0, mild pain = 1, moderate pain = 2, severe pain = 3

^b^The attending nurse who judged whether the patient need pain medication was blinded to the operative approach

^c^Administered if there was persistent or breakthrough pain

^d^A means the number of doses which is administered to the patients, R means the number of doses requested by the patients (as the maximum dosage of PCA was 4 mg morphine in 4 h and 10 mg in 24 h and the boluses were administered with a lockout interval of 10 min)

^e^If VAS > 4 despite morphine bolus

### Cosmetic results

Cosmetic appearance of the wound was assessed in 591 patients (Table 6). Standardized mean difference was calculated using a random-effects model since different scoring systems were used and wound cosmesis was assessed at varying moments. Overall better cosmetic results (three RCTs, *n *= 183) were reported after open hernia repair (SMD 1.21, 95% CI 0.50 to 1.92; *p* < 0.001; *I*^2^= 75%; Fig. 2F). Assessing only laparoscopic intracorporeal or extracorporeal suturing studies, these results persisted (Fig. 2F). No difference was found in cosmetic problems of the wound (i.e., hypertrophic scar, ugly scar, or stitch granuloma) between laparoscopic and open hernia repair (two RCTs, *n *= 333; OR 0.20, 95% CI 0.03 to 1.19; *p* = 0.08; *I*^2^= 0%; Fig. 1D).

**Table 6:** Cosmetic results

Author	Measurement	Measured by	Scoring system	Type of score	Timing of score	Wound score, mean (SD)/median (range)		Cosmesis problems, no. (%)	
						LH	OH	LH	OH
Celebi et al.	Recovery and wound appearance	Parents	70: fair 80: good 90: very good 100: excellent	Score 70–100	3 mo	89 (4.2)	78 (6.7)*	–	–
Chan et al.	Recovery and wound appearance	Parents	70: fair 80: good 90: very good 100: excellent	Score 70–100	7 d	95.4 (6)	90.2 (6)*	Hypertrophic scar: 1 (2.4)	Hypertrophic scar: 2 (4.8) Stitch granuloma: 1 (2.4)
Gause et al.	Wound appearance	Parents	1 (not satisfied) 2 3 (adequate) 4 5 (very satisfied)	Score 1–5	7 d	Uni: 4.9 (0.4) Bil: 4.9 (0.3)	Uni: 4.4 (0.8) Bil: 5 (0)	–	–
Inal et al.	–	–	–	–	–	–	–	–	–
Koivusalo et al.	Cosmetic result	Patients/parents, attending nurse and surgeon	0: unsatisfactory 1: satisfactory 2: good 3: excellent	Score 0–9	a) 6 mo b) 2 yr	a) 7 (3–9) b) 7 (5–9)	7 (3–9) 9 (5–9)	–	–
Saranga et al.	Scar cosmetics	Not clear	Good Excellent	Excellent/good Patients, no. (%)	Average 3.5 mo	Good: 0 (0) Excellent: 35 (100)	34 (100) 0 (0)	–	–
Shalaby et al.	Scar cosmetics	Parents	Ugly scar	Ugly scar Patients, no. (%)	> 6 mo	–	–	Ugly scar: 0 (0)	Ugly scar: 5 (4)*
Zhu et al.	–	–	–	–	–	–	–	–	–

*LH* laparoscopic hernia repair, *OH* open hernia repair, *SD* standard deviation, *d* days, *mo* months, *yr* year, *uni* unilateral, *bil* bilateral

*Significant difference between LH and OH group

## Discussion

In this systematic review and meta-analysis including evidence from eight RCTs representing 733 patients, we detected no differences in complication, recurrence, and MCIH rates between laparoscopic and open hernia repair. Unilateral operation time, length of hospital stay, and time to full recovery were also comparable. Laparoscopic hernia repair resulted in a mean reduction in operation time of 7.19 min; however, the clinical relevance of this difference is highly questionable. There is also evidence of substantial heterogeneity, which can only partially be explained by subgroup analysis.

Laparoscopic approaches can be subdivided into two subgroups according to the laparoscopic suturing technique (i.e., intracorporeal suturing and extracorporeal suturing) that was used. Compared to the open technique, less complications and shorter unilateral operation time (− 5.37 min) were noted after laparoscopic repair with extracorporeal suturing. Laparoscopic hernia repair with intracorporeal suturing resulted in shorter length of hospital stay (− 1.5 h). However, the clinical relevance of the latter two results is negligible.

The included studies had heterogeneous study populations, as two studies only included male children and study outcomes were assessed in different, partially overlapping age groups. However, in sensitivity analyses for complication rate, recurrence rate, and operation time, including only studies with low risk of bias regarding the selection of patients did not substantially change the effect estimates.

In this analysis, the total complication rate was not different between the LH and OH group. These results are supported by Yang et al., who also reported no significant differences in the incidence of hydrocele, wound infection, scrotal edema, erythema, and testicular atrophy [9]. However, the results of this meta-analysis should be interpreted with caution: as reflected in the degree of heterogeneity, the complications that were analyzed largely varied among the included studies. Thereby, selective reporting bias could not be assessed. In 2014, Esposito et al. reviewed 22 studies and concluded that there were less complications in the LH group (0.9% vs. 2.7%; *p *= 0.001) [10]. More recently, Feng et al. also found less postoperative complications (15 vs. 31 complications) and less major complications (i.e., scrotal edema, iatrogenic ascent of the testis, and testicular atrophy) in boys (4 vs. 14 complications) after laparoscopic hernia repair [11].

One of the benefits of laparoscopic hernia repair is the opportunity for intraoperative inspection of the contralateral groin. Approximately 40% of all children still have a CPPV after 2 years of age [20] and the estimated childhood risk of developing an inguinal hernia following the presence of a CPPV is 25–50% [21]. It can be assumed that contralateral exploration and closure of a CPPV, if present, can prevent development of an MCIH. In this meta-analysis, MCIH rate was not different between the two groups, although the included studies only assessed the presence of a CPPV during laparoscopy and performed subsequent closure of the PPV during the same session. None of the studies performed contralateral exploration and subsequent closure in the OH group. Koivusalo et al. did not close a laparoscopically detected asymptomatic CPPV in 12 patients (26%), but awaited subsequent development of MCIH. During 2 years of follow-up, MCIH developed in 3/12 (25%) patients, which resulted in a slightly higher overall MCIH rate following laparoscopic hernia repair (6.4%) compared to open hernia repair (4.8%) [16]. The latter results emphasize the controversy with respect to contralateral exploration as not every PPV necessarily develops into a clinically relevant hernia. Sensitivity analysis by excluding the study of Koivusalo et al. resulted in lower MCIH rates following laparoscopic repair. Still, these results should be interpreted with caution as the interval estimate of the odds ratio for metachronous contralateral hernia is very wide.

Previous studies repeatedly found a trend towards higher recurrence rates following laparoscopic hernia repair [8, 14, 16], especially in infants weighing three kg or less [22]. However, our meta-analysis shows that recurrence rates between LH and OH do not differ significantly. There was an inconsistent follow-up time that ranged from 24 h to 2 years, and again selective reporting bias could not be assessed. Additionally, as different laparoscopic techniques and advanced methods of minimally invasive closure of the inguinal hernia might affect the risk for recurrence, future analyses remain necessary.

There is insufficient evidence to draw definite conclusions regarding postoperative pain and wound cosmesis. Our results show that less administration of pain medication might be necessary after laparoscopic hernia repair with intracorporeal suturing and that open hernia repair results in better cosmesis. However, there is large conceptual heterogeneity among the included RCTs, since different protocols and scoring systems were used to assess postoperative pain and wound cosmesis. Again, selective reporting bias could not be assessed. Core outcome sets with unequivocal criteria and scoring systems are crucial to draw definitive conclusions about differences in postoperative pain and cosmetic appearance or problems. In this systematic review, we included all currently available RCTs (no language restrictions) in order to estimate treatment effects more precisely, and performed meta-analyses using a random-effects model. This study has several limitations. The quality of the included RCTs varied and there is a certain degree of clinical diversity in patient population (regarding gender and age) and intervention characteristics (e.g., a different number of trocars, varying suture materials, and different knotting techniques were used). Furthermore, reported outcomes and outcome definitions are not the same across studies. This is also reflected by the degree of heterogeneity and imprecision in the confidence intervals of the effect estimates. The certainty of the evidence according to GRADE is predominantly moderate for most outcome variables. Data to assess the risk of apneas and treatment-related healthcare costs were completely lacking.

In conclusion, no definite conclusions to decide on the superiority of one of either treatment strategies can yet be drawn from the available literature. Surgeons facile in both open and laparoscopic approaches can exploit relative advantages for each individual patient. For instance, it is clear that laparoscopic hernia repair offers more peroperative information on both groin areas compared to open surgery. Laparoscopic surgery might therefore be advantageous in cases of high diagnostic uncertainty, in children with high risk of MCIH development (especially infants as the risk increases with declining age) and in children with recurrent hernia repair; however, this treatment strategy does not simply fit all surgeons, and more importantly, all patients. We should take into account what exploitable advantages (or risks) we wish to invoke for a given patient in a given context, rather than simply choose one approach based on personal preference, ability, or clinically irrelevant superiority. Execution of large, prospective randomized trials that take into account all relevant outcome measures, the use of different laparoscopic and anesthetic techniques, and costs are inevitable to obtain homogenous results to decide on the superiority of one of either treatment strategy.

## Electronic supplementary material

Below is the link to the electronic supplementary material.

Supplementary material 1 (DOCX 41 kb)
