# Supplementary material for: Laparoscopic versus open pediatric inguinal hernia repair: state-of-the-art comparison and future perspectives from a meta-analysis
Source: Surg Endosc. 2019 Jul 17;33(10):3177–91. doi: 10.1007/s00464-019-06960-2 (PMC6722044; doi:10.1007/s00464-019-06960-2)
Supplement: Supplementary file 1 — Supplementary material 1 (DOCX 41 kb) [file 464_2019_6960_MOESM1_ESM.docx]

**Online Supplementary Material**

**Supplementary Material 1.** Search strategy

**Supplementary Material 2.** Data collected for studies included in the meta-analysis.

**Supplementary Material 3.** PRISMA flow diagram of the study selection and checklist.

**Supplementary Material 4.** Risk of bias summary and risk of bias graph.

**Supplementary Material 1. Search strategy**

Pubmed:

("hernia, inguinal"[MeSH] OR (("hernia"[tw] OR "hernias"[tw]) OR “herniorrhaphy“[tw] OR “herniotomy”[tw] AND "inguinal"[tw])) AND ("Child"[MeSH] OR "Child, preschool"[MeSH] OR "Young Adult"[MeSH] OR "Infant"[MeSH] OR "child"[tw] OR "children"[tw] OR "childhood"[tw] OR "schoolchild"[tw] OR "schoolchildren"[tw] OR "infant"[tw] OR "infants"[tw] OR "infancy"[tw] OR "boy"[tw] OR "boys"[tw] OR "boyhood"[tw] OR "girl"[tw] OR "girls"[tw] OR "girlhood"[tw] OR "youth"[tw] OR "youths"[tw] OR "toddler"[tw] OR "toddlers"[tw] OR "teen"[tw] OR "teens"[tw] OR "teenager"[tw] OR "Puberty"[Mesh] OR "puberty"[tw] OR "preschool"[tw] OR "pre school"[tiab] OR "pre-school"[tw] OR "juvenile"[tw] OR "young"[tw] OR "youngster"[tw] OR "youngsters"[tw] OR "schoolchild"[tw] OR "schoolchildren"[tw] OR "kid"[tw] OR "kids"[tw] OR "underage"[tw] OR "under age"[tw] OR "under aged"[tw] OR "puberal"[tw] OR "pubescent"[tw] OR "prepubescent"[tw] OR "prepuberty"[tw] OR "school age"[tw] OR "schoolage"[tw] OR "school ages"[tw] OR "Pediatrics"[Mesh] OR "Pediatrics"[tw] OR "Pediatric"[tw] OR "Paediatrics"[tw] OR "Paediatric"[tw]) AND ("laparoscopy"[MeSH] OR "laparoscopy"[tw] OR "laparoscopies"[tw] OR "laparoscopic"[tw] OR "minilaparoscopy"[tw] OR "minilaparoscopic"[tw]) AND ("Comparative Study" [Publication Type] OR compar*[tw] OR "open"[tw] OR "versus"[tw])

Embase:

(exp inguinal hernia/ OR (("hernia".mp. OR "hernias".mp.) OR "herniorrhaphy".mp. OR "herniotomy".mp. AND "inguinal".mp.)) AND (Exp Child/ OR exp young adult/ OR exp Infant/ OR "child".mp. OR "children".mp. OR "childhood".mp. OR "schoolchild".mp. OR "schoolchildren".mp. OR "infant".mp. OR "infants".mp. OR "infancy".mp. OR "boy".mp. OR "boys".mp. OR "boyhood".mp. OR "girl".mp. OR "girls".mp. OR "girlhood".mp. OR "youth".mp. OR "youths".mp. OR "toddler".mp. OR "toddlers".mp. OR "teen".mp. OR "teens".mp. OR "teenager".mp. OR exp Puberty/ OR "puberty".mp. OR "preschool".mp. OR "pre school".ti,ab. OR "pre-school".mp. OR "juvenile".mp. OR "young".mp. OR "youngster".mp. OR "youngsters".mp. OR "schoolchild".mp. OR "schoolchildren".mp. OR "kid".mp. OR "kids".mp. OR "underage".mp. OR "under age".mp. OR "under aged".mp. OR "puberal".mp. OR "pubescent".mp. OR "prepubescent".mp. OR "prepuberty".mp. OR "school age".mp. OR "schoolage".mp. OR "school ages".mp. OR exp Pediatrics/ OR "Pediatrics".mp. OR "Pediatric".mp. OR "Paediatrics".mp. OR "Paediatric".mp.) AND (exp laparoscopy/ OR "laparoscopy".mp. OR "laparoscopies".mp. OR "laparoscopic".mp. OR "minilaparoscopy".mp. OR "minilaparoscopic".mp.) AND (exp Comparative Study/ OR compar*.mp. OR "open".mp. OR "versus".mp.)

Cochrane:

(("hernia" OR "hernias" OR "herniorrhaphy" OR "herniotomy") AND "inguinal") AND ("Adolescent" OR "Young Adult" OR "Infant" OR "child" OR "children" OR "childhood" OR "schoolchild" OR "schoolchildren" OR "infant" OR "infants" OR "infancy" OR "boy" OR "boys" OR "boyhood" OR "girl" OR "girls" OR "girlhood" OR "youth" OR "youths" OR "toddler" OR "toddlers" OR "teen" OR "teens" OR "teenager" OR "Puberty" OR "puberty" OR "preschool" OR "pre school" OR "pre-school" OR "juvenile" OR "young" OR "youngster" OR "youngsters" OR "schoolchild" OR "schoolchildren" OR "kid" OR "kids" OR "underage" OR "under age" OR "under aged" OR "puberal" OR "pubescent" OR "prepubescent" OR "prepuberty" OR "school age" OR "schoolage" OR "school ages" OR "Pediatrics" OR "Pediatric" OR "Paediatrics" OR "Paediatric") AND ("laparoscopy" OR "laparoscopies" OR "laparoscopic" OR "minilaparoscopy" OR "minilaparoscopic") AND ("Comparative Study" OR compar* OR "open" OR "versus")

**Supplementary Material 2. Data collected for studies included in the meta-analysis.**

- Name of first author, year of publication
- Country of origin
- Study characteristics
  - Study design and time period
  - In- and exclusion criteria
  - Study population and patient characteristics
  - Method of selection
  - Primary and secondary outcome(s)
  - Follow-up specifications
- Intervention characteristics: (open or laparoscopic) inguinal hernia repair
  - Technical features of operation technique
  - Primary outcome parameter: operative (i.e. injury of spermatic vessels or spermatic cord, tuba lesions, bleeding and apnea) and postoperative complications (i.e. hematoma/scrotal edema, hydrocele, wound infection, iatrogenic ascent of the testis and testicular atrophy).
  - Secondary outcome parameters: duration of surgery, length of hospital stay, postoperative pain (pain scores and pain-medication requirement), return to full recovery, recurrence, metachronous contralateral inguinal hernia (MCIH) rate, and cosmetic results.

**Supplementary Material 3. PRISMA flow diagram of the study selection and checklist.**

Records identified through database searching
(n = 1086)

## Screening

## Included

## Eligibility

## Identification

Additional records identified through other sources
(n = 0)

Records after duplicates removed
(n = 674)

Records screened
(n = 674)

Records excluded
(n = 642)

Full-text articles assessed for eligibility
(n = 32)

Full-text articles excluded, with reasons
n = 21 not eligible
n = 3 no full-text

Studies included in qualitative synthesis
(n = 8)

Studies included in quantitative synthesis (meta-analysis)
(n = 8)

**Supplementary Material 4. Risk of bias summary and risk of bias graph.**

4.1 Risk of bias summary: review authors' judgements about each risk of bias item for each included study.

4.2 Risk of bias graph: review authors' judgements about each risk of bias item presented as percentages across all included studies.
